# Recent Progress on Copper‐Based Bimetallic Heterojunction Catalysts for CO_2_ Electrocatalysis: Unlocking the Mystery of Product Selectivity

**DOI:** 10.1002/advs.202309865

**Published:** 2024-04-18

**Authors:** Jiabao Huang, Xinping Zhang, Jiao Yang, Jianmin Yu, Qingjun Chen, Lishan Peng

**Affiliations:** ^1^ Key Laboratory of Rare Earths, Chinese Academy of Sciences Ganjiang Innovation Academy Chinese Academy of Sciences Ganzhou 341119 China; ^2^ School of Rare Earths University of Science and Technology of China Hefei 230026 China

**Keywords:** CO_2_ reduction, copper‐based bimetallic catalyst, electrocatalysis, heterojunction, product selectivity

## Abstract

Copper‐based bimetallic heterojunction catalysts facilitate the deep electrochemical reduction of CO_2_ (eCO_2_RR) to produce high‐value‐added organic compounds, which hold significant promise. Understanding the influence of copper interactions with other metals on the adsorption strength of various intermediates is crucial as it directly impacts the reaction selectivity. In this review, an overview of the formation mechanism of various catalytic products in eCO_2_RR is provided and highlight the uniqueness of copper‐based catalysts. By considering the different metals' adsorption tendencies toward various reaction intermediates, metals are classified, including copper, into four categories. The significance and advantages of constructing bimetallic heterojunction catalysts are then discussed and delve into the research findings and current development status of different types of copper‐based bimetallic heterojunction catalysts. Finally, insights are offered into the design strategies for future high‐performance electrocatalysts, aiming to contribute to the development of eCO_2_RR to multi‐carbon fuels with high selectivity.

## Graphical abstract

Recent breakthroughs in the development of copper‐based bimetallic heterojunction catalysts for the electrocatalytic CO_2_ reduction reaction (eCO_2_RR) are reviewed, with the goal of promoting CO_2_ conversion. Particular emphasis is placed on the significance of the adsorption strength of various metal combinations on intermediate products, as it greatly affects the catalyst's product selectivity.

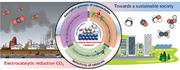

## 1 Introduction

**Figure 1:**
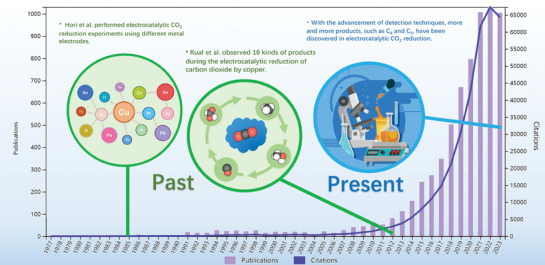
Retrieval results from the Web of Science using “electrocatalytic CO_2_ reduction” as the keyword, with several key historical milestones highlighted in the figure.

Modern societies heavily rely on fossil fuels for their energy needs, which has led to a significant increase in CO_2_ levels in the atmosphere, and disrupted the natural carbon cycle of the Earth. As our energy demands continue to grow, the severity of the energy crisis and environmental pollution also increases. Therefore, it is more important than ever to develop new renewable energy sources.^[^ ^1^, ^2^ ^]^ The electrocatalytic CO_2_ reduction (eCO_2_RR) process has garnered significant attention due to its facile controllability, mild operating conditions, and immense potential for sustainable energy conversion.^[^ ^3^, ^4^, ^5^ ^]^ This technology allows the conversion of the captured CO_2_ into valuable substances such as CO, formic acid, alcohols, or hydrocarbons. This process not only mitigates carbon emissions but also promotes the reuse of resources, thereby securing the energy supply.^[^ ^6^ ^]^ Implementing this technology represents a noteworthy stride toward sustainable production practices and a cleaner future. As shown in **Figure** **1**, there has been a steady increase in research related to electrocatalytic CO_2_ reduction in recent years. However, the electrocatalytic CO_2_ reduction technology is still in its early stages. The lack of efficient electrocatalysts with high activity, selectivity, and durability constraints the widespread industrial application of eCO_2_RR.^[^ ^7^, ^8^, ^9^ ^]^ As a result, researchers have set their sights on developing electrocatalysts that can offer high activity, selectivity, and stable CO_2_ reduction at low overpotential over an extended period.^[^ ^10^, ^11^, ^12^ ^]^

**Figure 2:**
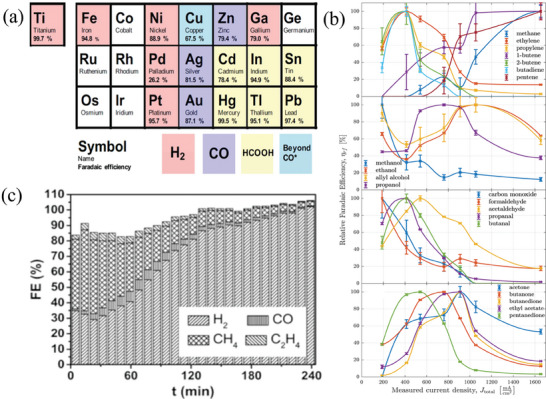
a) Results of CO_2_ reduction with different metal catalysts.^[^ ^14^ ^]^ Copyright 2017, Wiley‐VCH. b) Diversity of product distribution during CO_2_ electrocatalytic reduction on copper electrodes.^[^ ^24^ ^]^ Copyright 2023, Royal Society of Chemistry. c) Deactivation phenomenon occurring on copper electrodes.^[^ ^25^ ^]^ Copyright 2017, Wiley‐VCH.

The reduction of CO_2_ via eCO_2_RR on metallic materials is known to follow different reaction pathways with various reduction products. In a pioneering study by Hori et al., these metals showed four different reduction patterns, as illustrated in **Figure** **2**a: certain metals such as Cd, Pb, In, Bi, etc. mainly produced formate; Ag, Au, and Zn primarily yielded CO while Pt, Fe, Ni, Ti, Ga, Pd, etc. favors the competitive hydrogen evolution reaction to produce H_2_. However, Pd showed a unique behavior within this group of metals, with almost equal Faradaic efficiency for CO (FE_CO_) and H_2_ (FE_H2_) production.^[^ ^13^, ^14^ ^]^ Many researchers have conducted in‐depth studies based on these non‐copper‐based mono‐ or polymetallic catalysts to successfully electrocatalyst the reduction of CO_2_ to formate or CO.^[^ ^15^, ^16^, ^17^ ^]^ Compared to the other three types of metal‐based catalysts, copper‐based catalysts can deeply reduce CO_2_ to various hydrocarbons and organic matter containing oxygen, including C_2_ products like ethanol and ethylene and higher‐order products like propanol. The copper‐based catalysts exhibit higher catalytic efficiency in electrocatalytic CO_2_ reduction C‐C coupling, surpassing catalysts including PtRu,^[^ ^18^ ^]^ PdAu,^[^ ^19^ ^]^ NiGa,^[^ ^20^ ^]^ and MoS_2_.^[^ ^21^ ^]^ In addition, copper‐based catalysts have been considered as highly suitable catalyst materials for eCO_2_RR due to their cost‐effectiveness, widespread availability, and eco‐friendliness. However, until now, pure Cu catalysts have not been applied to actual industrial production due to issues regarding both selectivity and activity. The experimental results of Hori^[^ ^22^ ^]^ and Kuhl^[^ ^23^ ^]^ show that when pure Cu catalysts are employed in eCO_2_RR in 0.1 m KHCO_3_ solution, a total of 16 reduced products including C_1_ and C_2+_ products were produced at −0.7 to −1.2 V (vs RHE). Until recently, Rihm et al.^[^ ^24^ ^]^ demonstrated that, with the use of an ultra‐sensitive GC‐MS setup, it can even detect multiple C_5_ products during the CO_2_ electrocatalytic reduction process with copper‐based catalysts, their results are shown in Figure 2b. In the meantime, the copper electrode experiences severe deactivation during the reaction. Weng et al.^[^ ^25^ ^]^ have observed that when a pure copper electrode undergoes CO_2_ reduction in a 0.5 m KHCO_3_ solution at a potential of −0.96 V (vs RHE), the Faraday efficiency (FE) of hydrocarbons (methane and ethylene) decreases from 56% to 13% within 2 h, while the FE_H2_ increases to 80%. Furthermore, after 4 h, the catalyst almost completely loses its activity for eCO_2_RR, as shown in Figure 2c. This phenomenon is likely attributed to the deposition of carbonaceous substances on the surface of the copper electrode during the reaction.^[^ ^26^, ^27^, ^28^ ^]^

Research into maximizing the catalytic benefits of copper catalysts and overcoming their limitations in the catalytic process is an essential topic in CO_2_ electrocatalytic reduction systems.^[^ ^29^ ^]^ One effective approach is to incorporate a different metal into copper‐based catalysts to create bimetallic catalysts. Compared with monometallic catalyst systems, bimetallic catalysts can improve performance through various mechanisms, including ligand, strain, and geometric effects. By creating bifunctional active sites between adjacent metals, the activity of catalysts was enhanced while minimizing unwanted byproducts.^[^ ^30^, ^31^ ^]^ The constructed copper‐based bimetallic catalysts are primarily found in the form of heterostructures. Benefited by the unique electron transfer and interfacial synergy, copper‐based bimetallic heterojunction catalysts have been extensively studied and widely applied in many fields.^[^ ^32^, ^33^, ^34^ ^]^ For instance, Liu et al.^[^ ^35^ ^]^ synthesized bimetallic phosphide nanosheets on copper foam containing a significant amount of Cu_3_P/Ni_2_P heterogeneous interface. This catalyst‐based water electrolyzer only required a low cell voltage of 1.56 V@10 mA cm^−2^. Li et al.^[^ ^36^ ^]^ constructed a Cu/Zn bimetallic oxide S‐type heterojunction that can activate the peroxymonosulfate (PMS) system well, which can be used for catalytic wastewater degradation. Ganesh et al.^[^ ^37^ ^]^ prepared a bimetallic Cu_2_O‐CeO_2_/C heterojunction catalyst, which formed an interfacial heterojunction that enhanced the catalyst's absorption of visible light and was used to improve the photocatalytic activity in the Sonogashira cross‐coupling reaction.

This review focuses on the differences in adsorption intermediates and product selectivity of different types of copper‐based bimetallic heterojunctions for CO_2_ electrocatalytic reduction. This is the main difference between our review and other similar reviews, and it is also the current similar reviews are missing. In this review, we first briefly introduce the formation mechanism of various products in the eCO_2_RR process. Although there is still debate about the formation mechanisms of these products, they are closely related to the types and strengths of adsorbed intermediates on the catalyst surface during the reaction process, leading to different product orientations for different metals in the eCO_2_RR process. Based on this, we categorize numerous metals into four types based on their adsorption preferences:

Ni, Pt, and Pd, which preferentially adsorb *H
In and Sn, which preferentially adsorb *OCOH
Ag, Au, and Zn, which preferentially adsorb *COOH
Cu, which moderately adsorbs various intermediates, including *CO, *OCOH, *COOH, and so on.

Then, we discuss and analyze the changes in the adsorption process of intermediates and the resulting products when the first three categories of metals are combined separately with copper‐based catalysts to form copper‐based bimetallic heterojunction catalysts. Finally, we propose possible reaction trends based on different metal combinations to gain a better understanding of the role of copper‐based bimetallic heterojunctions in eCO_2_RR. These findings offer valuable insights and perspectives for designing future catalysts. By understanding the unique properties and behaviors of these copper‐based bimetallic heterojunctions, researchers can develop more effective strategies to improve their performance.

## 2 CO_2_ Electrocatalytic Reduction Process

### 2.1 CO_2_ Electrocatalytic Reduction Mechanism

The eCO_2_RR process is a complex and multi‐step reaction that involves several crucial stages. First, the CO_2_ molecule is adsorbed and activated on the catalyst surface. Next, various active intermediates are formed through electron transfer and/or proton migration. Finally, the reduction products are desorbed from the electrode surface and diffused into the electrolyte. Although the electrode potentials for generating different reduction products are relatively low (as shown in **Table** **1**), an applied voltage of up to −1.9 V is required for CO_2_ activation. That is because CO_2_ is a very stable linear molecule, with two delocalized bonds between carbon and oxygen atoms, rendering it much less reactive and a high thermodynamic energy barrier. Breaking the C = O bond requires considerable energy ≈750 kJ mol^−1^.

**Table 1:** Reduction potentials for the generation of different products.

Reaction equation	Product	Standard hydrogen electrode potential [eV]
CO_2_+2H^+^+2e^−^	CO+H_2_O	−0.10
CO_2_+2H^+^+2e^−^	HCOOH+H_2_O	−0.12
CO_2_+6H^+^+6e^−^	CH_3_OH+H_2_O	0.03
CO_2_+8H^+^+8e^−^	CH_4_+2H_2_O	0.17
2CO_2_+12H^+^+12e‐	C_2_H_5_OH+3H_2_O	0.09
2CO_2_+12H^+^+12e^−^	C_2_H_4_+4H_2_O	0.08
2H_2_O	O_2_+4H^+^+4e^−^	1.23
2H^+^+2e^−^	H_2_	0.00

Two types of activation of *CO_2_ adsorbed on the catalyst surface have been identified: the first is activation induced by proton‐electron co‐transfer mechanism (CPET), and the second is characterized by sequential proton transfer and electron transfer mechanism (SPET).^[^ ^38^ ^]^ These mechanisms compete, producing a range of reactive intermediates, including *COOH, *OOCH, *OCHO, and *CO, etc. The selectivity of the reaction is determined by the relative binding strength of these reactive intermediates as well as *H on the catalyst surface.

**Figure 3:**
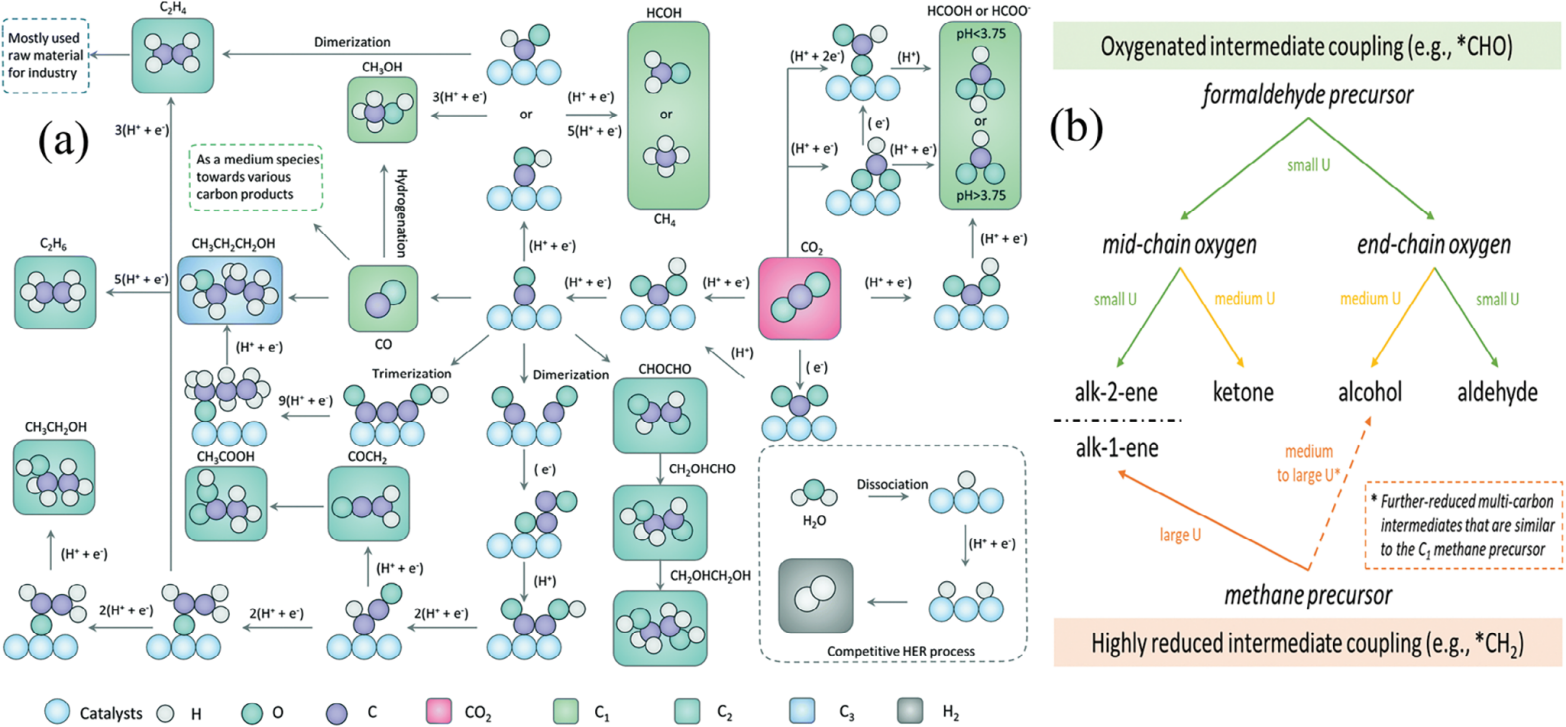
a) Possible roadmaps of eCO_2_RR toward various value‐added products. Different mechanistic controversies when electrocatalytic reduction of CO_2_ occurs.^[^ ^57^ ^]^ Copyright 2022, Royal Society of Chemistry. b) General hypotheses regarding the eCO_2_R mechanism.^[^ ^24^ ^]^ Copyright 2023, Royal Society of Chemistry.

In general, the *HCOO intermediate and its conversion to produce the *OCHO intermediate are considered more favorable for formic acid formation. While *COOH can also produce formic acid, the *CO intermediate that arises from it is crucial in generating CO and other hydrocarbon products. The *CO intermediate is considered vital for forming C_2_ and C_2+_ products, meanwhile, it can also generate C_1_ hydrocarbon products by producing *CHO intermediates,^[^ ^39^, ^40^, ^41^, ^42^ ^]^ including CH_3_OH, HCOH, and CH_4_.^[^ ^43^ ^]^ Despite significant advances in understanding catalytic reactions, many questions still need to be answered regarding forming C_2_ and C_2+_ products. For example, the reaction process of ethanol and ethylene in C_2_ products includes *CH_2_ carbon dimerization, *CO dimerization, *CO‐COH coupling, and other mechanisms.^[^ ^44^, ^45^ ^]^ For more advanced C_2+_ reduction products, such as C_3_ products like propanol,^[^ ^46^, ^47^, ^48^ ^]^ acetone,^[^ ^49^ ^]^ and propanal,^[^ ^50^ ^]^ their reaction pathways have not yet been thoroughly explored.^[^ ^51^, ^52^, ^53^ ^]^ With the development of various detection techniques, more and more reaction products and intermediates, including C_4_ and C_5_ products, have been discovered.^[^ ^24^ ^]^ This underscores that there remain many aspects of the CO_2_ electrocatalytic reduction mechanism that require further research.^[^ ^54^, ^55^, ^56^ ^]^ Some possible reaction processes are shown in **Figure** **3**.^[^ ^57^ ^]^

Reaction intermediates must often be strongly adsorbed and highly covered on the catalyst surface to achieve C‐C bond coupling. This results in an increase in the energy barrier required for the reaction. Besides, there are other obstacles to the electrocatalytic reduction of CO_2_. The slow kinetic rate of the multiple electron transfer processes involved in the reaction limits its speed. This slow kinetic process requires a high overpotential to accelerate the reaction rate, causing a high loss rate and low electrical energy utilization. Electrocatalytic CO_2_ reduction reactions typically occur in aqueous systems, where H_2_O provides H^+^ for the reaction due to its good conductivity and low cost. However, since the potential of the hydrogen evolution reaction is similar to that of the reduction reaction, competing evolution reaction (HER) is unavoidable in aqueous systems. The occurrence of competing reactions reduces the selectivity of carbon‐based products. It affects the coverage of *CO on the catalyst surface, which is not conducive to the dimerization of *CO close to each other.

The CO coverage on the catalyst surface is not only closely related to the properties of the electrocatalytic material itself but also jointly affected by various factors such as temperature,^[^ ^58^ ^]^ pressure, pH,^[^ ^59^ ^]^ surfactant^[^ ^60^ ^]^ or other organic additives,^[^ ^61^ ^]^ and the electrolyte anion and cation species^[^ ^62^, ^63^ ^]^ in the electrolyte. Therefore, the researchers attempted to achieve precise regulation of the catalyst surface *CO coverage by adjusting these factors:

1) Constructing Tandem Catalysts: The catalyst needs to effectively convert the surface‐adsorbed CO_2_ into *CO to ensure a high CO coverage on the catalyst surface, but the ability of Cu to activate CO_2_ and produce *CO is relatively weak. Introducing a second metal to construct a bimetallic heterojunction and achieve tandem catalysis has become an effective way to solve this problem. By using the second component to produce *CO and compensate for the insufficient production of *CO by Cu‐based catalysts, it can help achieve C‐C coupling.^[^ ^64^ ^]^ This will also be covered in detail in a subsequent section.
2) Adjusting the Surface pH: pH is an important factor that affects the *CO/*H coverage in the system, directly determining whether the reaction pathway is the dimerization or protonation of *CO. When the pH of the system is low, the main adsorbed product is *H, which will lead to severe HER reactions and the production of CH_4_ as the CO_2_ reduction product. On the other hand, in a high pH environment, the interaction between OH^−^ and CO can enhance the adsorption of CO on Cu, helping to increase the *CO coverage on the catalyst surface and promoting the dimerization of CO. This can help improve the ability of copper‐based bimetallic catalysts to produce C_2+_ products. However, it should be noted that under alkaline conditions, OH‐ may react with dissolved CO_2_ to form HCO_3_^−^ and CO_3_^2−^ ions, reducing the concentration of CO_2_ on the catalyst surface and affecting the rate of CO_2_RR.^[^ ^65^ ^]^
3) Increasing the Concentration or Partial Pressure of Carbon Dioxide: It has been proven that increasing the concentration or partial pressure of carbon dioxide on the catalyst surface can improve the *CO coverage and promote the production of C_2+_ products.^[^ ^66^ ^]^
4) Potential Control: By adjusting the potential, the adsorption processes of *CO and *H can be precisely controlled. As the potential increases, more *CO will be produced on the surface of the copper‐based catalyst, thereby increasing the *CO coverage. In addition, increasing pressure can also improve the concentration of CO by promoting its dissolution in the electrolyte, further optimizing the catalytic effect.^[^ ^67^ ^]^

### 2.2 Classification of Different Metals

**Figure 4:**
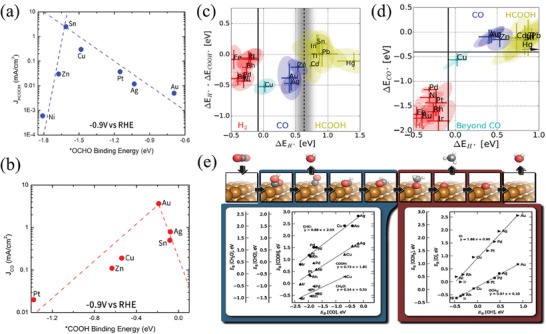
a) Volcano plot of *OCHO binding energy as a descriptor of HCOO^−^ partial current density.^[^ ^68^ ^]^ Copyright 2017, American Chemical Society. b) Volcano plot of *COOH binding energy as a descriptor of CO partial current density.^[^ ^68^ ^]^ Copyright 2017, American Chemical Society. c) Metal classification using *H adsorption energy and ΔE_*H_‐ΔE_*COOH_ as reaction descriptors.^[^ ^14^ ^]^ Copyright 2017, Wiley‐VCH. d) Metal classification using *H adsorption energy and *CO adsorption energy as reaction descriptors^[^ ^14^ ^]^ Copyright 2017, Wiley‐VCH. e) The linear relationship between different key intermediate adsorption energies during CO_2_ electrocatalytic reduction to CH_4_.^[^ ^70^ ^]^ Copyright 2012 American Chemical Society.

For the four groups of metals mentioned above, in the electrocatalytic reduction of CO_2_: (1) the first group of metals Ni, Pt, Pd, etc., which tend to HER reaction thus produce H_2_; (2) the second group of metals Sn, In, etc., which produce formic acid or formate; (3) the third group of metals Ag, Au and Zn, which produce CO; and (4) the fourth group consists solely of Cu, which are capable of producing a variety of C_1_‐C_3_ products, including carbon monoxide, methane, ethanol, ethylene, propanol, etc. This categorization of metals based on their reaction products is vital in electrocatalysis, as it helps researchers predict the behavior of different metal combinations in various electrochemical reactions. Essentially, the selectivity of the catalytic product is intricately connected to both the type and intensity of the intermediates bound during the reaction. Here we mainly discuss the effects of the following intermediates during the reaction process: *H, *CO, *COOH, *OOCH, and *OCHO. By using the binding energy of *COOH and *OCHO as descriptors for producing CO and HCOO^−^, the relevant current density volcano plot shows that the second category of metals is more thermodynamically favorable for adsorbing *OCOH, as shown in **Figure** **4**a. The third category of metals is more inclined toward adsorbing *COOH, as shown in Figure 4b. Based on this information, it can be inferred that among the various categories of metals listed, the optimal choices for CO and HCOOH formation are Au and Sn, respectively.^[^ ^68^ ^]^

By considering the binding energy of *H on metal surfaces, we can observe different behaviors for different types of metals under the corresponding reduction potential, as shown in Figure 4c. The first type of metal is located in the hydrogen underpotential deposition (H_upd_) region, where hydrogen can be deposited on the metal surface; For the second type of metal, there is a difference between the binding energy of *H and *COOH (ΔE_*H_ – ΔE_*COOH_) that is greater than 0. This means that the metal surface has little or no coverage of hydrogen; On the other hand, for the third type of metal, the ΔE_*H_ – ΔE_*COOH_ values are all less than 0. This indicates that *H can be adsorbed on the catalytic surface sites that can generate *COOH intermediates, while the metal surface has enough *H adsorption capacity to undergo the HER reaction. The positive and negative values of ΔE_*H_ – ΔE_*COOH_ can explain why the Faraday efficiency for the metals that generate CO is generally lower than that for generating HCOOH, with the latter even reaching almost 100% EF_HCOOH_. The fourth type of metal, which is only represented by Cu, can achieve multiple‐carbon products. If the binding force between the catalyst and CO is too weak, the catalyst surface does not have sufficient *CO coverage to carry out coupling reactions; On the other hand, if the binding is too strong, it will lead to a high activation energy barrier for the coupling reaction and make it difficult to achieve the hydrogenated intermediates. Cu catalysts can meet these stringent conditions, with moderate affinity for CO, different from metals such as Pt and Ni, which are more susceptible to CO poisoning, or metals such as Au and Ag, which desorb before CO protonation. Density functional theory (DFT) calculation results in Figure 4d indicate that Cu is the only metal with a negative *CO adsorption energy and a positive *H adsorption energy, which means that Cu can bind *CO without depositing hydrogen. However, in the CO_2_ reduction reaction, Cu has a moderate *CO adsorption energy and a moderate binding energy for most intermediates, which contributes to various competing reaction pathways and complex products over pure Cu catalysts.^[^ ^69^ ^]^

From a thermodynamic perspective, single‐metal catalysts, including Cu catalysts, are affected by the linear relationship between the adsorption energies of continuous intermediate species in the CO_2_ electro‐reduction process, which leads to the inability to independently control the adsorption energy of key intermediates. As a result, the activity and selectivity of single‐metal catalysts are inevitably limited. For example, the correlation between the adsorption energy of different intermediates in the CO_2_ electrocatalytic reduction and the formation of the CH_4_ process is described in detail in Figure 3e. This correlation also affects the competition with the HER reaction. The adsorption energy of *H is linearly proportional to the adsorption energy of COOH* and CO*, making it difficult to decrease the adsorption energy of *COOH without increasing the yield of H_2_.^[^ ^70^ ^]^

### 2.3 Advantages of Constructing Heterostructures

The introduction of a second metal can effectively address the challenges associated with Cu‐based catalysts. This strategy disrupts the proportional relationship between different intermediates and enhances the reaction selectivity toward desired products. Consequently, researchers have sought to engineer catalyst structures to expand their range of applications.^[^ ^71^, ^72^, ^73^ ^]^ Heterostructures have gained prominence due to the functional characteristics of multiple components,^[^ ^74^, ^75^, ^76^ ^]^ primarily attributed to the interaction of different materials at the contact interfaces. These interfaces are not merely physical subdivisions; they also possess distinctive electronic and catalytic properties that provide active regions for the reaction.^[^ ^77^, ^78^, ^79^ ^]^ Due to the differing energy band structures of the materials that constitute the heterostructure, the energy bands will adjust accordingly when these materials come into contact to accommodate a new equilibrium state. Specifically, before the two materials make contact, it is possible for one side of the material to have a higher Fermi energy level than the other. Once contact is established, to reach an equilibrium state, the Fermi energy level will change, resulting in electron transfer from the material with the higher Fermi energy level to the one with the lower, generating an electric field. This process continues until the Fermi energy levels on both sides of the materials are equalized. After the formation of a heterojunction, the energy bands on the side with the initially higher Fermi energy level will bend downwards to form a region of negative charge, while the energy bands on the side with the lower Fermi energy level will bend upwards to form a region of positive charge. This bending of energy bands and the creation of a built‐in electric field affect the charge density and potential distribution on both sides of the interface, profoundly impacting the electrochemical performance of the heterostructure electrode. For the application of eCO_2_RR, heterostructures present advantages in two aspects.

1) Interface Effect: The adsorption energy of intermediates is paramount in dictating the efficacy and selectivity of electrocatalytic CO_2_RR reactions. In the dynamic interface of heterogeneous structures, the positive and negative regions near the interface display unique reactivities toward various intermediates due to the intricate interplay of energy bands and electron redistribution.^[^ ^80^ ^]^ This allows for the precise modulation of adsorption energies at different interfacial locations, fostering desired reaction pathways while hindering others. This regulatory ability makes heterogeneous structures a powerful tool for optimizing the eCO_2_RR process. For instance, the in‐situ reconstructed Sn/SnO_x_ interface has significant adsorption strength for *OOCH and *COOH intermediates.^[^ ^81^ ^]^ As a result, it reduces the limit potential of HCOOH and increases CO potential at the interface, consequently exhibiting substantial HCOOH selectivity in the Cu‐Sn/SnO_x_ electrocatalysts.
2) Tandem Catalysis: Tandem catalysis encompasses simultaneous or continuous multi‐step reactions, as it engages multiple active sites.^[^ ^82^, ^83^, ^84^ ^]^ Effective coupling between different active components in the heterostructure can avoid intermediate product purification and separation while reducing energy consumption.^[^ ^85^ ^]^ Tandem catalysis frequently occurs in the CO_2_ to C_2+_ products process, catalyzed by bimetallic catalysts like Ag and Au.^[^ ^86^ ^]^ Subsequent metals absorb the *CO intermediate produced by Ag and Au, for C‐C dimerization. The performance of Ag and Au catalysts can be maximized by adjusting the concentration and bonding strength of the *CO intermediate, by varying their composition and structure.

Heterostructures have demonstrated other effects that enhance CO_2_ conversion to different products. The activation of CO_2_ molecules during the C_1_ product formation benefits from the synergistic effect. On one hand, Lewis's acid‐base pairs and hydrogen bonds stabilize and bend CO_2_ molecules, converting them into C_1_ products.^[^ ^87^ ^]^ On the other hand, C‐C coupling produces C_2+_ products, a more complex process than C_1_ product formation. The micro‐environment effect can increase local CO coverage, promoting the C‐C dimerization reaction on the catalyst surface. The complicated structure of heterogeneous catalysts promotes eCO_2_RR as multiple effects coexist within them.^[^ ^88^ ^]^

Despite similarities in composition, the design philosophies of heterostructures and composite materials are fundamentally different.^[^ ^89^, ^90^, ^91^ ^]^ The design of composites often focuses on combining the favorable properties of different materials without deeply exploring how the intrinsic characteristics of each component material affect the overall performance. In contrast, the design of heterostructures is based on a profound understanding of the internal properties of the materials, such as the energy band structure, arrangement, and differences in Fermi energy levels, allowing for precise control and utilization of electron and ion behavior.^[^ ^92^ ^]^

Therefore, when designing heterostructures for the electrocatalytic reduction of carbon dioxide, the key lies in the precise control and utilization of electronic and ionic behavior within the materials, coupled with a deep understanding of the electronic/ionic environments in hybrid catalysts to optimize catalytic performance. By increasing the number of heterogeneous interfaces, it is possible to take full advantage of the performance enhancement brought about by the interfaces to maximize the favorable changes occurring in the heterogeneous materials^[^ ^78^ ^]^ and to achieve efficient catalysis of the CO_2_ reduction reaction. This approach not only improves catalytic efficiency but may also enhance the selectivity of target products through the precise design of heterogeneous interfaces, offering new strategies and possibilities for the electrocatalytic reduction of carbon dioxide.

## 3 *H Adsorption‐Type Metals

When metals such as Ni, Pt, and Pd are used in CO_2_ electrocatalysis, their strong affinity toward *CO and *H can lead to severe CO poisoning during the reaction and a high tendency to undergo HER reaction. Therefore, when this kind of metal is combined with copper, how to use the *H adsorption ability of this kind of metal to hydrogenate the intermediate and promote the formation of *CO without poisoning is a problem that needs to be solved in the application of this kind of metal.

### 3.1 Cu‐Ni Heterojunction Catalysts

When nickel (Ni) based catalysts are used alone in CO_2_ electrocatalytic reduction, the products' selectivity and the catalyst's size have a great relationship. The research results of Wang^[^ ^93^ ^]^ and Li^[^ ^94^ ^]^ show that the smallest Ni single‐atom catalysts are more likely to undergo CO_2_ electrocatalytic reduction reactions to produce CO than to undergo HER reactions. However, as the Ni‐based catalyst's size increases, the HER reaction tendency also increases.^[^ ^95^ ^]^

**Figure 5:**
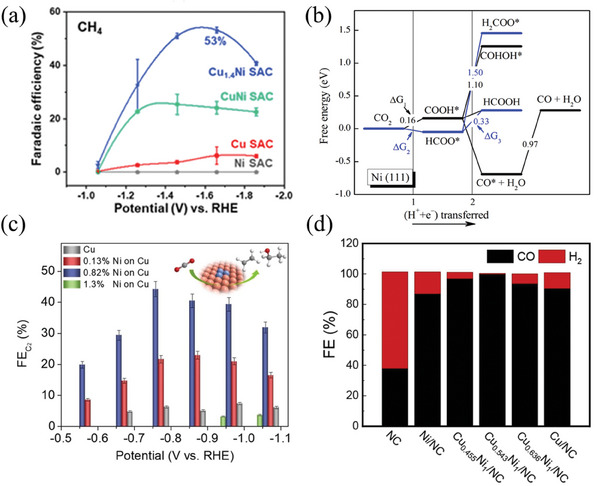
a) the methane selectivity of Cu‐Ni bimetallic catalysts at different potentials.^[^ ^88^ ^]^ Copyright 2021, American Chemical Society. b) the free energy diagram of CO_2_ reduction to HCOOH on Ni (111) surface at 0 V versus RHE.^[^ ^103^ ^]^ Copyright 2017 American Chemical Society. c) the CO selectivity obtained by changing the percentage of surface Ni atoms on Cu at different voltages.^[^ ^104^ ^]^ Copyright 2021, Elsevier. d) the CO selectivity of CuNi alloy catalysts with different atomic ratios at −0.6 V.^[^ ^102^ ^]^ Copyright 2022, Elsevier.

Some research teams^[^ ^96^ ^]^ have conducted theoretical calculations on the simplest flat‐layered Cu‐Ni heterostructure model. They suggest that Ni is unsuitable as Cu's bottom or middle layer material for CO_2_ electrochemical reduction. The calculation results show that due to the compressive strain effect of Ni, the Cu (1–3 ML, ML is a deposition pseudo single layer) ‐Ni (211) layered structure is not conducive to the adsorption of *COOH and *CHO intermediates, but shows stronger adsorption toward *CO and *OH. However, if Cu is used as the bottom layer of Ni, the opposite result is presented, and this process is also affected by the thickness of the Ni layer. Yoshihara et al.^[^ ^97^ ^]^ deposited Ni on a Cu foil to prepare a Cu‐Ni stacked bilayer electrode and found that a thinner Ni stacked electrode can selectively form hydrocarbons (mostly CH_4_, with a small amount of C_2_H_4_) during CO_2_ electrocatalytic reduction, with a current efficiency of 56.2%. In contrast, a thicker Ni‐deposited electrode generates hydrogen. Todoroki et al.^[^ ^95^ ^]^ used molecular beam epitaxy (MBE) to synthesize Ni/Cu (111) bimetallic substrates under ultra‐high vacuum (UHV) conditions. At a thickness of 0.1‐ML, the total FE was slightly higher than that of Cu (111). Although the FE_CH4_ decreased, the FE_CO_ increased. When the Ni layer thickness increased to 1‐ML, the total Faraday efficiency was lower than that of Cu (111). Li et al.^[^ ^98^ ^]^ synthesized a neighboring Cu‐Ni bimetallic atomic catalyst (Cu_1.4_Ni SAC). Only a single Cu atom acted as a catalytic site for the C_1_ reduction pathway, showing high selectivity for CH_4_. At −1.66 V (vs RHE), the FE_CH4_ was 53%, as shown in **Figure** **5**a.^[^ ^99^ ^]^

Tan et al.^[^ ^100^ ^]^ implemented a hydrothermal‐annealing method to build CuNi alloy nanoparticles embedded in nitrogen‐doped carbon skeletons (Cu_1.0_Ni_1.0_/NC). These nanoparticles effectively converted CO_2_ into CO under the conditions of 0.5 m KHCO_3_ and −0.6 V (vs RHE), with a Faradaic efficiency for CO (FE_CO_) of 94.5%. In a similar vein, Yi et al.^[^ ^101^ ^]^ grew Cu‐Ni alloy nanoparticles (Cu_1.0_Ni_1.0_/NCNT) as catalysts on nitrogen‐doped carbon nanotubes using a similar synthesis method, achieving an FE_CO_ of 94.8% under the same conditions. Yang et al.^[^ ^102^ ^]^ optimized the ratio of CuNi alloy nanoparticles and obtained a Cu_0.543_Ni_1_/NC structure, which significantly improved the FE_CO_ to 99.7% under the same test conditions, as shown in Figure 5d.

Zhao et al.^[^ ^103^ ^]^ calculated two pathways for the generation of formic acid on the Ni (111) surface and found that the Ni (111) surface would be poisoned by *CO during the generation of formic acid, making it unable to produce formic acid. However, they believed that Ni deposited on a copper bottom layer (Cu/Ni) can selectively generate HCOOH through a reaction pathway that does not produce CO, with a reaction overpotential of −0.14 V, as shown in Figure 5b.^[^ ^104^ ^]^ Jitaru et al.^[^ ^105^ ^]^ synthesized Cu‐Ni nanotubes by electrochemical deposition in an alumina film, showing 50% FE_HCOOH_. However, the structure was unstable during the electrolysis process and collapsed, so it could only be electrolyzed for a short time. Yang et al.^[^ ^106^ ^]^ synthesized nano‐porous Cu/Ni oxide composite materials (CuNiOCs), and when the Cu/Ni ratio was 3:1 (Cu_3_NiOCs), the FE_HCOO‐_ could reach 95.9%, and the reaction overpotential was only 0.37 V. The outstanding electrocatalytic performance of this catalyst is mainly attributed to its porous structure, the synergistic effect of two oxides, large electrochemical surface area, and low charge transfer resistance. Even after 25 h of electrolysis, it remains stable.

In other reports, Cu‐Ni catalysts with different structures have also shown potential for synthesizing C_2_ products. As shown in Figure 5c, Zhang et al.^[^ ^104^ ^]^ uniformly dispersed Ni atomic clusters on the surface of copper nanowires (CuNW) rich in defects, and the modified Ni only existed on the Cu surface. When the surface Ni atomic percentage was 0.82%, the FE_C2+_ at −0.77 V (vs RHE) was 44%, with the highest FE_C2H5OH_ of 16%. The highest FE_C2H4_ was 24% at −0.97 V. In a 1.0 M KOH electrolysis cell, the Faraday efficiency of C_2_ products can be further increased to 62%, and the FE_C2H5OH_ was 22.9% at −0.88 V, and FE_C2H4_ was 31.8%. The calculation results show that the energy barriers of all C_1_ intermediates (*CO_2_, *COOH, *CO) are significantly reduced under Ni modification. Bader charge analysis shows that the adjacent Cu obtains additional electrons from Ni, reducing the potential kinetic barrier of C‐C coupling. Tomiko M et al.^[^ ^107^ ^]^ used oxide‐derived Cu‐Ni alloy nanoparticles with a size of 10 nm, which showed higher C_2_ compound selectivity (especially ethanol and ethylene) than pure Cu. When the Ni content was between 0.32 at%, the Faraday efficiency of C_2_H_4_ formation could reach 22%. This Cu‐Ni catalyst is a mixture of metal Cu, Cu O, metal Ni, and Ni O. The reaction mechanism may be that adjacent Ni and Ni‐O species change the electronic structure of the Cu active center, or Ni species inhibit the formation of HCOOH and C_2_ products. Huang et al.^[^ ^108^ ^]^ designed and synthesized a 1D composite fiber structure Ni(CNFs)@Cu(CNFs) (with Ni/C as the core, Cu/C as the shell, and CNFs as carbon nanofibers), which separated Ni and Cu metal clusters by a carbon skeleton to form a series of catalysts. The core‐shell structure inhibits the release of H_2_, completes the directional mass transfer of CO_2_ from Ni/C to Cu/C, and finally converts it into ethanol. The FE_C2H5OH_ at −1 V (vs RHE) is up to 18.2%. Zhang et al.^[^ ^109^ ^]^ prepared Nitrogen‐doped 3D nanoporous graphene‐loaded CuNi nanoparticles (CuNi@C/N‐npG), exhibit excellent ethanol product selectivity at −0.78 V, with a FE_C2H5OH_ reaching 84%. DFT calculations have demonstrated that the strong metal support interaction (Ni‐N‐C) can effectively modulate the surface electronic structure, facilitate electron transfer, stabilize active sites on the surface, and ultimately achieve controllable transitions of intermediate reaction products.

### 3.2 Cu‐Pt Heterojunction Catalysts

In recent years, there have been relatively few studies on single‐metal Pt‐based catalysts for CO_2_ electrocatalytic reduction.^[^ ^110^ ^]^ Experimental results show that the CO_2_ reduction activity on the Pt plane is: Pt (111) < Pt (100) ≪ Pt (110) < Pt (210). Fan et al.^[^ ^111^ ^]^ showed that Pt (210) has a higher density of (110) step sites and exhibits higher CO_2_ electrocatalytic activity on different Pt stepped surfaces. Hoshi et al.^[^ ^22^ ^]^ demonstrated that a twisted Pt stepped surface can enhance CO_2_ electrocatalytic activity, and the rate of CO_2_ reduction increases with increasing twisted atomic density. However, in most cases, CO_2_ reduction reactions on the Pt surface will be poisoned by the CO generated, blocking the active sites on Pt. In contrast, the Faraday efficiency of the HER reaction occurring on the Pt surface is close to 100%, and hydrogen production has high selectivity.^[^ ^112^ ^]^

**Figure 6:**
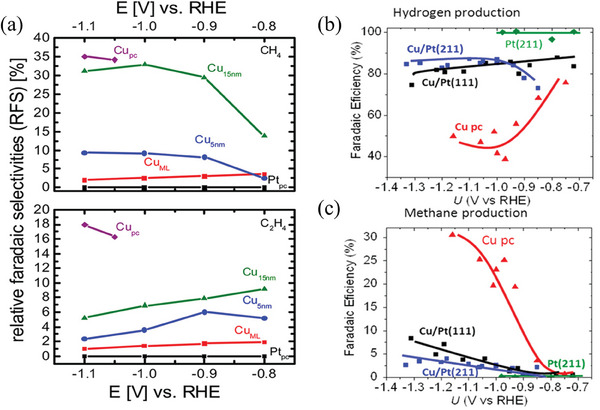
(a) Relative Faradaic selectivity (RFS) of CH_4_ and C_2_H_4_ produced on Pt polycrystalline with different thicknesses of deposited Cu during CO_2_ electroreduction in 0.1 m KHCO_3_.^[^ ^113^ ^]^ Copyright 2013, American Chemical Society. b) FE_H2_ and c) FE_CH4_of polycrystalline Cu, Pt (211), Cu/Pt (211), and Cu/Pt (111) after 15 min of CO_2_‐saturated 0.1 m KHCO_3_ electrolysis.^[^ ^112^ ^]^ Copyright 2013, American Chemical Society.

Platinum's low CO_2_ electrocatalytic activity is also reflected in the synthesized Cu‐Pt bimetallic heterostructures, and there are a few examples of such structures used for CO_2_ electrocatalytic reduction. Todoroki et al.^[^ ^95^ ^]^ showed that when the thickness of the Pt layer on the Cu (111) metal plate reaches 0.1 ML, the CO_2_ electrocatalytic reduction reaction on the Cu (111) surface is significantly inhibited. Ana et al.^[^ ^112^ ^]^ deposited a Cu coverage layer on the Pt (111) and Pt (211) surfaces, and there was no significant difference in activity between the Cu/Pt (111) and Cu/Pt (211) surfaces during CO_2_ electrocatalytic reduction, and both highly tended to undergo the HER reaction. The methane production efficiency was also much lower than that of polycrystalline Cu itself,^[^ ^113^ ^]^ as shown in **Figure** **6**a,b. They attributed the reaction selectivity to the Cu/Pt surface recombination induced by the *CO intermediate. The strong interaction between *CO and Pt during the reaction process forces the Cu atoms on the Cu/Pt surface to approach each other and expose part of the Pt surface. During the change process, the Cu coverage layer will be subjected to a certain degree of tensile strain from Pt, making the surface more strongly bound to *CO and other intermediates, reducing the CO_2_ electrocatalytic reduction activity of the Cu coverage layer. This dynamic change process indicates that the system containing Pt is unstable in the electrochemical reduction process of CO_2_.

However, if the Cu layer is thick, it can inhibit the exposure of Pt atoms and show higher selectivity for hydrocarbon products. By changing the thickness of the Cu coverage layer loaded on Pt, the product selectivity of the CO_2_ reduction reaction can be controlled. That is consistent with the results of Liu et al.^[^ ^114^ ^]^ who calculated the adsorption capacity of different intermediate layers in different Cu‐Pt heterostructures. Under the interaction of the tensile strain and ligand effect exhibited by Pt, the Cu (1‐2 M)‐Pt layered structure exhibits stronger adsorption on four intermediates (*CO, *COOH, *COH, *CHO) than the pure Cu surface, but when Cu is stacked to three layers, the adsorption capacity of Cu (3 M)‐Pt on *CO intermediates is lower than that of the single Cu surface. This theory was confirmed in the experiment of Reske et al.^[^ ^115^ ^]^ Although the catalytic activity of CO_2_ reduction increases with the thickness of the copper layer, it is always lower than that of pure copper catalysts. Therefore, they believe that platinum is an ineffective hydrocarbon production catalyst. Tang et al.^[^ ^116^ ^]^ studied the surface segregation phenomenon in Cu‐Pt nanoalloy particles and proposed that this phenomenon would be enhanced with particle size, surface openness, or overall Cu content. At the same time, surface segregation will continue to compete with bulk alloying, and the corresponding ordered trend will eventually transfer to the Pt‐rich side or large particle size. In addition, the Cu‐Pt ratio in the Cu‐Pt alloy nanoparticles greatly influences the catalytic activity. Increasing the Cu content can effectively improve the selectivity for CO_2_. However, when the Cu content is too high, the adsorption of *H is insufficient, which limits the reaction activity, and the most suitable proportion needs to be found. Guo et al.^[^ ^117^ ^]^ studied Cu‐Pt alloy nanocrystals. When the Cu content was 0.759, the Faraday efficiency of the electrocatalytic CO_2_ reduction to CH_4_ in 0.5 m KHCO_3_ at room temperature was about 21%. Zhao et al.^[^ ^118^ ^]^ synthesized a Cu_85_Pt_15_ alloy nanocube with a high copper concentration, and the CO Faraday efficiency can reach more than 20%.

### 3.3 Cu‐Pd Heterojunction Catalysts

**Figure 7:**
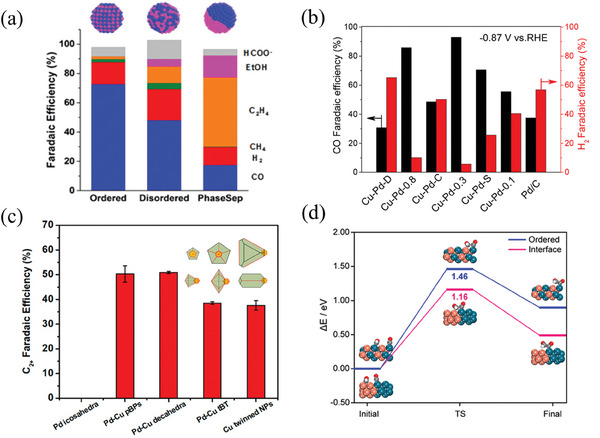
a) Reduction selectivity of Cu‐Pd catalysts with different mixing modes.^[^ ^124^ ^]^ Copyright 2016, American Chemical Society. b) The CO and H_2_ Faradaic efficiency of morphologically distinct bimetallic copper–palladium nanoalloys at −0.87 V.^[^ ^131^ ^]^ Copyright 2018, American Chemical Society. c) FE_C2_ of three Pd‐Cu Janus nanocrystals: pentagonal bipyramid, decahedron, and truncated bipyramid during the reaction.^[^ ^130^ ^]^ Copyright 2020, American Chemical Society. d) Kinetic barriers of the coupling between *CO and *CHO on the ordered Cu−Pd and the interface within the phase‐separated Cu− Pd alloys.^[^ ^125^ ^]^ Copyright 2021, American Chemical Society.

Palladium has a very special position in this group of metals. According to the data of Hori et al., Pd has similar CO and H_2_ Faraday efficiencies (−0.81 V).^[^ ^13^ ^]^ The unique catalytic activity of Pd catalysts is attributed to different active phases produced at different reduction potentials. Pd can not only adsorb H atoms on the metal surface but also absorb them into the lattice to form palladium hydride,^[^ ^119^, ^120^ ^]^ and the behavior of Pd lattice absorbing and releasing hydrogen atoms is reversible.^[^ ^121^ ^]^ The electrode potential will affect the H coverage status on the Pd surface. As the potential decreases, the H coverage rate on the Pd catalyst surface gradually decreases, and different active phases are generated on the rich and poor hydrogen surfaces.^[^ ^122^, ^123^ ^]^ By forming a heterostructure of Cu and Pd, the chemical adsorption strength of intermediates on the catalyst surface can be adjusted through geometric and electronic effects, thereby achieving the best activity and selectivity for eCO_2_RR. The studies of Ma^[^ ^124^ ^]^ and Li^[^ ^125^ ^]^ both show that the selectivity of bimetallic Cu‐Pd catalysts depends on the geometric effect. Cu‐Pd nanoalloy particles with different atomic mixing modes (ordered, disordered, phase‐separated) exhibit different CO_2_ reduction selectivity, part of the reaction products can be seen in **Figure** **7**a.

Ordered Cu‐Pd nanoparticles are conducive to producing C_1_ products, with most research focusing on CO and CH_4_. Mun et al.^[^ ^126^ ^]^ synthesized monodisperse Cu‐Pd nanoalloy particles with different ratios using the colloid method. The 1:1 Cu‐Pd ratio synthesized Cu‐Pd nanoalloy particles can achieve 87% CO Faraday efficiency at −0.9 V (vs RHE). DFT results show that the binding of Pd and Cu increases the energy barrier of the CO protonation stage, and the produced CO can be released before hydrogenation. The Pd_85_Cu_15_/C alloy nanocrystal catalyst synthesized by Yin et al.^[^ ^127^ ^]^ exhibits a CO Faraday efficiency of 86% at −0.89 V. However, as the Cu‐Pd catalyst transitions from the ordered phase to the disordered phase, the FE_CH4_ increases, possibly due to the higher intermediate coverage on the disordered Cu‐Pd surface.^[^ ^124^ ^]^ Xie et al.^[^ ^128^ ^]^ synthesized a new Cu‐Pd heterostructure derived from CuCl‐PdO_x_ hexagonal microporous plates. The Cu/Pd ratio in the Cu‐Pd heterostructure can be adjusted by changing the concentration of the precursor Pd salt. The Cu‐Pd heterostructure synthesized when the precursor Pd salt concentration was 0.68 mm achieved a CH_4_ catalytic effect of 32% EF (−1.25 V). DFT calculation results also show that the hollow sites in the Pd region of the Cu‐Pd heterostructure can stabilize the CO intermediate, selectively reduce the energy demand for CH_4_ production, transfer electrons to *CO, and provide binding sites for H^+^. Wang et al.^[^ ^129^ ^]^ synthesized a series of quasi‐cubic Cu_3_Pd_x_N nanocrystals to convert CO_2_ to methane. When the voltage was −1.25 V, the FE_CH4_ of Cu_3_Pd_0.62_N reached 57.5%. Zhu et al.^[^ ^130^ ^]^ synthesized different‐shaped Pd‐Cu bimetallic nanocatalysts. As shown in Figure 7c, the flower‐shaped Pd_3_Cu (FL‐Pd_3_Cu) achieved an EF_CO_ of 82.1% at −0.9 V, while the rhombic dodecahedron Cu_3_Pd (CRD‐Cu_3_Pd) was more conducive to the production of CH_4_, and the FE_CH4_ reached 40.6% at −1.2 V. Chen et al.^[^ ^131^ ^]^ synthesized spherical CuPd nanocatalysts, achieving an EF_CO_ of 93% at a potential of −0.87 V as shown in Figure 7b. In contrast, the branched CuPd nanocatalysts demonstrated an EF_H2_ of 65.2%. However, compared with the phase‐separated Cu‐Pd catalyst, the ordered state is more conducive to producing C_1_ products.

The phase‐separated Cu‐Pd system is considered more conducive to producing C_2_ products,^[^ ^124^, ^125^ ^]^ and the system has a lower d‐band center, showing weaker *CO binding and lower spatial hindrance than Cu. Band charge shows that the carbon atoms adsorbed on the segregation interface inside the phase‐separated alloy exhibit more significant atomic charge differences, which is conducive to generating C‐C bonds. CO is preferentially adsorbed in the Pd region, and when the *CO coverage reaches the limit, it overflows from the Pd region to the Cu region. In the Cu region, *CO and *CHO coupling occurs at a lower energy barrier, as shown in Figure 7d. Ma et al.^[^ ^124^ ^]^ observed that the FE_C2_ of phase‐separated Cu‐Pd nanoparticles in the experiment could reach more than 60%. Feng et al.^[^ ^132^ ^]^ prepared Cu‐Pd/CP (carbon paper) by electrodepositing, an efficient catalyst for reducing CO_2_ to C_2_H_4_. At −1.2 V (vs RHE), the FE_C2H4_ can reach 45.2%. Lyu et al.^[^ ^130^ ^]^ synthesized Cu‐Pd Janus nanocrystals of different shapes. At −1.0 V (vs RHE), the FE_C2+_ species of pentagonal bipyramid, decahedron, and truncated bipyramid reached 50.3%, 51.0%, and 38.5%, respectively, respectively mainly including ethanol and ethylene.

It is worth mentioning that Pd is the only metal material that can reduce CO_2_ to formate at near‐zero overpotential. That is due to the unique CO_2_ reduction mechanism of hydrogenated palladium. CO_2_ does not directly bind to the Pd surface but contacts with hydrogenated palladium, and formate is generated directly without the CO_2_ activation process under high potential electron transfer. Therefore, besides the main C_1_ products (CO and CH_4_) mentioned above, some Cu‐Pd bimetallic heterostructures are also used to produce HCOOH. Şahin et al.^[^ ^133^ ^]^ prepared Cu_50_Pd_50_/C cathode material by microwave‐assisted polyol method, selectively converting CO_2_ to HCOOH, and the Faraday efficiency reached 60% at −0.72 V. Wang et al.^[^ ^134^ ^]^ found that Cu_20_Pd_80_/C prepared by the wet synthesis process can achieve 60% FE_HCOO‐_, while the same proportion of catalyst prepared by H_2_ thermal reduction can only observe 7.73%, which may be due to the production of H_2_ during the reaction. In the Cu‐Pd heterostructure synthesized by Xie et al.^[^ ^128^ ^]^ using a higher Pd precursor salt concentration (1.36 mm), the FE_HCOOH_ reached 64% at −0.9 V. Takashima et al.^[^ ^124^ ^]^ deposited a Cu‐modified layer (UPD‐Cu/Pd) on Pd nanoparticles at low potential, with a particle size of 5 nm, and the FE_HCOO‐_ reached 84%. They believe that the charge transfer of Pd to Cu improves the catalyst's tolerance to CO.

When forming a bimetallic heterojunction structure with Cu and metals (Ni, Pt, Pd) that have a tendency to adsorb *H, the *CO adsorption ability of the three metals shows a trend of Pd<Ni<Pt (while *H adsorption is the opposite), which is consistent with the activity of the resulting Cu‐metal heterojunction structure in CO_2_ electrocatalytic reduction. When the content of Ni, Pt and other metals is relatively low, the adsorption process of Cu toward intermediates can be changed, leading to a more favorable reaction direction and more production of C_1_ products. With the appropriate metal ratio or special catalyst structure, C_2_ reactions can also occur, which is particularly evident in catalysts represented by Pd. Currently, Bimetallic heterojunction catalysts consisting of such metals and copper have certain limitations, more or less, mainly due to the shortcomings in the performance of Cu‐Ni and Cu‐Pt, as well as the cost restrictions imposed by precious metals such as Cu‐Pt and Cu‐Pd. In this review, we enumerate these studies to comprehensively elucidate the characteristics of copper‐based bimetallic heterostructures with different metal combinations. We draw lessons from successful cases and also learn from less successful examples, which undoubtedly contribute to the development of research. These efforts provide more choices for the application of copper‐based bimetallic heterostructures and also indicate potential directions for incorporating more non‐precious metal elements and constructing ternary and quaternary catalysts in the future. We eagerly anticipate the early realization of these applications, injecting new vitality into the field of catalyst development.

## 4 *OOCH and *OCHO Adsorption Type Metals

Metals such as Sn and In can effectively inhibit the competitive hydrogen evolution reaction in the eCO_2_RR process due to their high hydrogen evolution overpotential. These metals can convert CO_2_ into *OOCH, and the adsorption energy of *OOCH and *COOH on the Cu surface is similar. Therefore, the selectivity of bimetallic catalysts closely depends on the interaction between the two metals. In addition, Sn and In are easy to oxidize, and the surface of oxidized catalysts may show different intermediate adsorption energies.

### 4.1 Cu‐Sn Heterojunction Catalysts

Sn is located at the peak of the *OCHO kinetic volcano diagram, making it an effective electrocatalyst material for producing HCOOH. Because of its high reactivity, Sn easily oxidizes, forming the Sn‐O bond which facilitates CO_2_ adsorption and results in good CO_2_RR activity. As a non‐noble metal, Sn is non‐toxic and is often used to form heterojunction catalysts with Cu, making it a highly promising material for use in practical applications in CO_2_ reduction reactions.^[^ ^135^, ^136^ ^]^

**Figure 8:**
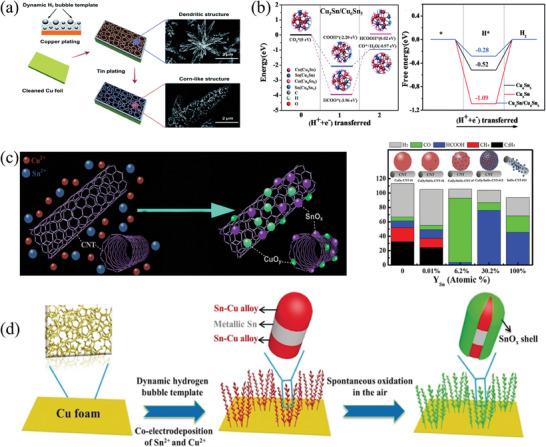
a) The formation of a 3D core‐shell porous Cu@Sn catalyst.^[^ ^137^ ^]^ Copyright 2019, Royal Society of Chemistry. b) The intermediate energy calculation diagram of CO and HCOOH on the surface of Cu_3_Sn/Cu_6_Sn_5_ heterostructure and the free energy diagram of HER on the three catalysts.^[^ ^138^ ^]^ Copyright 2019, Royal Society of Chemistry. c) The growth of CuO_y_/SnO_x_ nanoparticles on CNT and the relationship between the structure and product distribution of Cu/SnO_x_‐CNT catalyst.^[^ ^141^ ^]^ Copyright 2017, American Chemical Society. d) Synthesis route of 3D layered Sn‐Cu catalyst.^[^ ^143^ ^]^ Copyright 2020, Wiley‐VCH.

Compared to single metal catalysts, Cu‐Sn bimetallic catalysts can attain superior HCOOH Faraday efficiency during CO_2_ electroreduction. The 3D core‐shell porous electrode prepared by Hou et al.^[^ ^137^ ^]^ by the two‐step electrodeposition method displayed a high selectivity of 100% for HCOOH (**Figure** **8**a). The secondary dendritic structure offers ample *OOCH active sites, thanks to its significant specific surface area. The adsorption strength of *H, which is consumed during the reaction process of Cu‐Sn catalyzed CO_2_ reduction to HCOOH, influences the selectivity of HCOOH. Kang et al.^[^ ^138^ ^]^ found that an overabundance of *H adsorption on Cu_6_Sn_5_ and Cu_3_Sn obstructed active sites. Despite this, Cu_3_Sn/Cu_6_Sn_5_ heterostructure catalysts produced higher‐quality HCOOH (FE = 82%) as well as having improved electrocatalytic reduction of CO_2_ due to moderate adsorption of *H intermediates (Figure 8b). The interaction between Sn and Cu can change the electronic structure and promote the protonation of CO_2_*^−^. Li et al.^[^ ^139^ ^]^ investigated the mechanism of the production of HCOOH from the perspective of calculation. The Sn/Cu interface in Sn@Cu served as an active center for CO_2_ adsorption and activation. The Sn atom gave electrons as well as caused the d‐band center to shift negatively, changing the adsorption strength of *OOCH intermediates. Protonation of CO_2_*^−^ constituted the rate‐determining step in the reaction.

For the Cu/SnO_x_ heterojunction catalysts, CO is the predominant reaction product. As an illustration, the 3D dendritic core‐shell Cu/CuOx‐SnOx catalysts performed particularly well, with a selectivity of 94% and high activity for CO generation in an aqueous solution.^[^ ^140^ ^]^ This excellent performance arose from their unique foam morphology and dendritic nanostructures, which increased the concentration of CO_2_ near the active site and improved the long‐distance transmission of CO_2_. Wang et al.^[^ ^141^ ^]^ studied Cu/SnO_x_‐CNT catalysts and found that CO was mainly generated at Cu sites (Figure 8c). With the assistance of adjacent SnO_x_ sites, electrons were transferred from the oxide to the Cu nanoparticles at the Cu/SnO_x_ interface. Increased electron density on the Cu site reduced the adsorption of CO intermediates, thereby facilitating the production of CO. Simultaneously, Sn atoms altered the adsorption sites on the Cu surface, impeding the adsorption of *H intermediates and therefore hindering the production of H_2_ and HCOOH. Studies have demonstrated that the catalytic performance is related to the relative content of Cu and Sn within the catalyst. In their study, Li et al.^[^ ^142^ ^]^ compared the performance of Cu/SnO_2_ and annealed A‐Cu/SnO_2_. The Faraday efficiency of CO is highest, and the formation of HCOOH is slow in the A‐Cu/SnO_2_ catalyst, indicating a similar Cu‐Sn synergistic effect at different SnO_2_ deposition times. In contrast, the selectivity of Cu/SnO_2_ catalyst shows significant change with SnO_2_ deposition. With an increase in deposition time, the selectivity shifts from CO to HCOOH. The varying catalytic properties are attributed to the effect of the relative content of Cu atoms in the SnO_2_ layer on the charge density at the Cu‐Sn interface.

Some reports have shown that interfacial reconstruction can occur in Cu‐Sn catalysts with specific compositions, leading to improved Faraday efficiency of C_1_ products. Ye et al.^[^ ^143^ ^]^ designed a 3D hierarchical structure catalyst on Cu foam, with heterostructures constructed using a highly conductive Cu‐Sn alloy as the metal core and SnO_x_ as the shell (Figure 8d). During the eCO_2_RR process, the Sn_2.7_Cu core‐shell structure experienced destroyed surface free energy balance, with internal Sn atoms spontaneously migrating into the SnO_x_ shell, forming the Sn/SnO_x_ interface. As a result of this interfacial reconstruction, the Sn_2.7_Cu catalysts exhibited excellent catalytic performance with a C_1_ Faraday efficiency of about 96.0% at 0.93 V (vs RHE). According to DFT calculations, the reconstructed Sn/SnO_x_ interface was responsible for the high Faraday efficiency of C_1_ products, in comparison to Sn (211) and SnO_2_ (110). This reconstruction weakened the binding of *OOCH and *COOH intermediates. Furthermore, the energy barrier for the formation of *H at the interface was significantly improved, leading to significant inhibition of competitive HER.

### 4.2 Cu‐In Heterojunction Catalysts

Although In‐based catalysts exhibit high selectivity for HCOOH, this process occurs at a potential greater than −1.0 V (vs RHE), and the resulting energy efficiency is notably low, making it unsuitable for practical application. Moreover, the stability of In in air is slow to the point where it may transform into In_2_O_3_ and In(OH)_3_ phases during eCO_2_RR. Therefore, researchers believe that the catalytic active phases of indium are likely to be In^0^, In_2_O_3_, and In(OH)_3_.^[^ ^144^, ^145^, ^146^ ^]^ As a result, current studies on Cu/In heterojunction catalysts mostly focus on these three components.^[^ ^147^, ^148^, ^149^ ^]^

**Figure 9:**
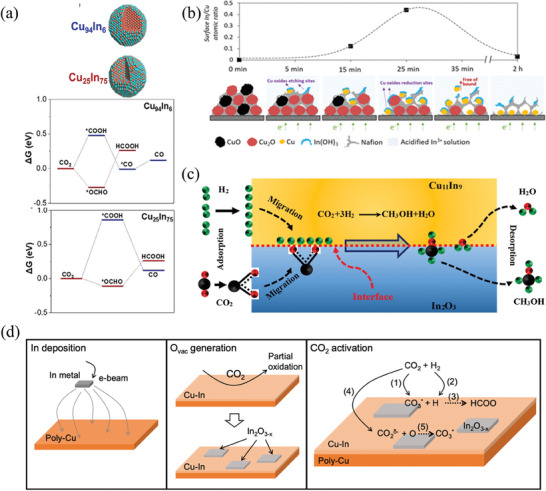
a) The schematic diagram of Cu_94_In_6_ and Cu_25_In_75_ and the free energy diagram of CO_2_ reduction to CO and HCOOH at 0 V vs RHE.^[^ ^150^ ^]^ Copyright 2019, Wiley‐VCH. b) In/Cu atomic ratio diagram on the electrode surface during 0–2 h ESP.^[^ ^153^ ^]^ c) The reaction mechanism of CO_2_ hydrogenation to CH_3_OH over Cu_11_In_9_.^[^ ^140^ ^]^ Copyright 2019, Elsevier. d) Phase evolution and CO_2_ hydrogenation reaction process on the Cu‐In catalyst.^[^ ^155^ ^]^ Copyright 2021, Elsevier.

The pure In phase can achieve high HCOOH selectivity only under high overpotential conditions. The addition of an appropriate amount of Cu decreases the energy barrier for the reaction and improves the electrocatalytic activity of CO_2_. In this process, the synergistic effect between In and Cu can also improve the current density and stability. One study reported that Cu_25_In_75_ nanoparticles, prepared using a co‐precipitation method, exhibited excellent performance at low overpotential.^[^ ^150^ ^]^ At the potential of −0.7 V (vs RHE), the Faraday efficiency of HCOOH reached 84.1%, which remained above 70% even when the potential was reduced to −0.5 V (vs RHE), while FE_H2_ was less than 5%. DFT calculations showed that the high HCOOH selectivity of this catalyst was attributed to the low binding strength of *OOCH on Cu_25_In_75_ and the low energy barrier of protonation (**Figure** **9**a). Extra coordination of unsaturated atoms on the surface acts as active sites, enhancing HCOOH selectivity. Dendritic Cu‐In catalysts with a high specific surface area were prepared by Shao et al.^[^ ^151^ ^]^ through two‐step electrodeposition. The Cu‐In catalysts achieved a Faraday efficiency for HCOOH of 87.4% when electrodeposited for 30 minutes, substantially surpassing the Faraday efficiencies of In foam (57%) and Cu foil (19.2%) under the same condition. Moreover, the catalyst maintained an efficiency of over 80% for formic acid production for over twelve hours.

The Cu‐In interface improves *COOH adsorption strength for higher CO selectivity, as reported by Luo et al.^[^ ^152^ ^]^ with their in‐situ electrochemically prepared Cu nanowire catalysts with deposited In. The highly dispersed In particles create a significant Cu‐In interface, improving CO selectivity over a wider potential range, with Faraday efficiency close to 95%. In the 60 h stability test, the total current density and Faraday efficiency did not fluctuate greatly. Mechanistic analysis indicated that the excellent selectivity was primarily due to Cu‐In interface sites, which reduced the Gibbs free energy changes of *COOH and the desorption energy barriers of CO. Moreover, the interaction between Cu and In hindered the hydrogen adsorbate reaction on the Cu‐In surface. As reported by another study, a combination with a gas diffusion electrode was spontaneously deposited electrochemically to synthesize Cu‐In catalysts.^[^ ^153^ ^]^ The ratio of In to Cu on the catalyst surface changed during deposition, and eventually, an In(OH)_3_/Cu_x_O heterostructure was deposited (Figure 9b). The resulting CO Faraday efficiency was about 90% due to the Cu‐In interaction.

The In_2_O_3_ phase plays a pivotal role in catalyzing CO_2_ hydrogenation to CH_3_OH by cyclically creating and eliminating oxygen vacancies. However, weak H_2_ adsorption capacity limits the CO_2_ conversion rate, which can be remedied by adding Cu to the In electrode surface to adjust H_2_ adsorption capacity according to Shi et al.^[^ ^154^ ^]^ found that at the reaction temperature of 350 °C, their catalysts demonstrated unusually high CH_3_OH selectivity (FE = 80.5%) compared with other Cu‐based catalysts. This was attributed to the formation of Cu_11_In_9_ through segregation during the reduction process, which adjusted the Cu electronic structure and enhanced H_2_ adsorption capacity. Additionally, the Cu_11_In_9_/In_2_O_3_ interface functioned as an active site for CH_3_OH synthesis. Mechanistic studies showed that on the Cu_11_In_9_ surface, H_2_ dissociated to *H, whereas CO_2_ primarily adsorbed onto In_2_O_3_ oxygen vacancies (Figure 9c). Li et al.^[^ ^155^ ^]^ performed further research into Cu/In surface activation and CO_2_ hydrogenation, detailing phase transitions of Cu and In and the CO_2_ hydrogenation process diagram (Figure 9d). The findings reinforced Shi's interfacial catalytic mechanism and offered new insight into how CO_2_ is primarily present as CO_3_* during activation on the Cu/In interface.

After the doping of Cu into metals like Sn and In, which possess high adsorption capabilities for *OOCH and *OCHO, the catalysts primarily produce C_1_ products, with heterostructures being formed later. The relative content of Cu and the metals is crucial in determining the selectivity of the heterojunction catalyst. Catalysts with high Sn and In content exhibit excellent Faradaic efficiencies of HCOOH, whereas doping Cu into metals promotes CO formation. Additionally, uncontrollable variables greatly influence the eCO_2_RR selectivity. Metal oxides and hydroxides, such as SnO_x_, In_2_O_3,_ and In(OH)_3_, are routinely produced under electrocatalytic conditions due to their electrophilic properties. Intermediates' binding strengths differ significantly on these substances. In addition to the compounds formed during electrocatalysis, catalyst interface evolution also plays a crucial part in eCO_2_RR by modifying the activation energy of the intermediate reaction.

## 5 *COOH Adsorption‐Type Metals

Ag, Au, and Zn activate *COOH to *CO, and when combined with Cu, can enhance local CO concentration and promote C‐C coupling. According to the Sabatier principle, the best catalyst surface has excellent adsorption strength for key reaction intermediates. Weak *CO adsorption strength on the catalyst surface results in instability after a certain coverage, and yields CO as the main product. Conversely, high *CO coverage reduces its binding energy due to repulsion between adsorbates, promoting the formation of other products.

### 5.1 Cu‐Ag Heterojunction Catalysts

The desorption thermodynamic potential energy of the *CO intermediates is less than zero on Ag's surface, making it difficult to reduce or polymerize further. Hence, the Ag electrode is inclined to reduce CO_2_ to CO. However, the overpotential of this reaction is near the standard reduction potential of CO_2_ conversion to CO, necessitating high energy drive. This drawback limits Ag's application in CO_2_ electrochemical reduction.^[^ ^156^ ^]^ Cu binding changes the catalyst's electronic structure, allowing easy electron transfer to activated CO_2_ and enhancing CO_2_ reduction activity.^[^ ^157^ ^]^

**Figure 10:**
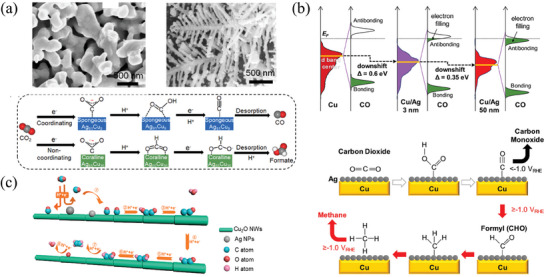
a) SEM images of sponge‐like Ag_91_Cu_9_ and coral‐like Ag_65_Cu_35_ catalysts and the CO_2_ reaction pathways on their surfaces.^[^ ^158^ ^]^ Copyright 2020, Elsevier. b) CO binding energy diagram for the three catalysts and diagram of CO and CH_4_ formation mechanism on Cu‐Ag catalyst with Cu shell pore size of 3 nm.^[^ ^160^ ^]^ Copyright 2020, Elsevier. c) Mechanism of CO_2_ reduction to C_2_H_4_ on Cu‐Ag catalyst.^[^ ^161^ ^]^ Copyright 2019, American Chemical Society.

A Cu‐Ag nanoparticle catalyst with adjustable C_1_ product selectivity was achieved by precisely adjusting the Cu‐Ag atomic ratio in one study (**Figure** **10**a), with a Faraday efficiency of 91.8% to generate HCOOH achieved by Ag_65_Cu_35_ at −1.0 V (vs RHE).^[^ ^158^ ^]^ The excellent performance of the Cu‐Ag catalyst is attributed to both electronic and geometric effects. The increase in alloying degree with the introduction of Cu shifts the d‐band center upward, leading to increased binding strength of the intermediate *COOH and *CO, and making CO desorption more challenging from an electronic standpoint. Moreover, the atomic arrangement of the Ag_65_Cu_35_ site produces a synergistic effect between the Ag and Cu atoms, resulting in both atoms acting as active centers in the reaction. On the Ag_65_Cu_35_ (111) surface, the negative formation energy of *OOCH enables CO_2_ reduction to HCOOH more easily from a geometric standpoint.

Under certain conditions, Cu/Ag catalysts produce CH_4_ as the main product of the CO_2_ reduction reaction. Cheng et al.^[^ ^159^ ^]^ conducted a study on the production process of C_2_H_4_ and found that *CO is preferentially adsorbed on the edges of the Cu island. The decrease in size of the edges of the Cu island was suggested to promote the exposure of low‐coordination surface sites, resulting in the combination of low‐coordination sites with CO intermediates and hindrance of movement and dimerization of *CO. This process promoted the formation of CH_4_. Changing the applied potential of CO_2_ reduction reactions in special catalysts can lead to the production of CH_4_. Dong et al.^[^ ^160^ ^]^ synthesized Cu/Ag catalysts with varying Ag layer thicknesses. Among them, the catalyst with an Ag layer thickness of 3 nm displayed superior bifunctional catalytic activity with high CO selectivity at moderate overpotential. Upon applying a potential of −1.2 V, the selectivity of the electrode to CH_4_ was significantly increased (FE_CO_‐FE_CH4_ = −53.1%). The reason for this selective conversion was revealed by DFT calculation, which showed that the binding energy of CO on the Cu/Ag 3 nm surface is lower than the overpotential of CH_4_ generation (Figure 10b). Consequently, CO escapes when the applied energy reaches its binding energy, and only when the energy is sufficient for CO hydrogenation, does the production of CH_4_ become the dominant pathway.

Cu‐Ag catalysts facilitate the C‐C coupling at the interface through the spillover effect, leading to the formation of multi‐carbon products. This tandem catalytic mechanism was explained by Gao et al.^[^^]^ using Operando Raman Spectroscopy (Figure 10c). The vibration of *CO adsorbed at the Cu site and the Raman peaks of CO adsorbed on the Ag surface can be observed on the Cu‐Ag catalyst. Although both Ag and Cu are active sites for CO production, the weak binding between Ag and CO causes CO to migrate to the surface of Cu where dimerization occurs at a sufficiently high potential. The Cu‐Ag nanowire catalyst exhibited a high Faraday efficiency of 76% for C_2+_ products. Comparing the CO spillover efficiency of Ag to Cu on different catalysts, the results show that the effective spillover of CO is the key to significant improvement of catalyst performance. In a previous study, Huang and colleagues^[^ ^162^ ^]^ discovered that the transfer of electrons from Cu to Ag at the interface enhances the adsorption of CO on the surface of Cu. Compared to single metal nanoparticles, the Faraday efficiency of Ag_1_‐Cu_1.1_ ND for C_2_H_4_ was enhanced by 3.4 times.

Studies have focused on combining CO and Cu in the spillover phenomenon to improve the selectivity of C_2+_ products. The increase of the surface coverage of *CO can greatly promote the adsorption of *CO, and the confinement effect of the catalyst pore structure influences the selectivity by changing the *CO concentration. Recently, Zhong et al.^[^ ^163^ ^]^ found a relationship between the pore size of the Cu shell in the Ag@Cu catalysts and the selectivity of C_2+_ products. The catalysts with an average pore size of 4.9 nm exhibited the highest selectivity of C_2+_ products, with a selectivity ratio of C_2+_/C_1+_ of 5.1. FEM simulation revealed that the 4.9 nm Cu shell exhibited the strongest confinement effect, resulting in a significant increase in the local concentration of CO and promotion of C‐C coupling that generates multi‐carbon products.

### 5.2 Cu‐Au Heterojunction Catalysts

Among all metals, Au exhibits the highest adsorption strength for *COOH and thus displays very high CO selectivity. However, the overpotential required for CO_2_ reduction driven by Au is higher even than that of Ag. As such, Cu and its oxides are often used for modification. Compared to single‐metal catalysts, the potential of Cu‐Au synergistic catalysis for CO_2_ reduction is much lower.

**Figure 11:**
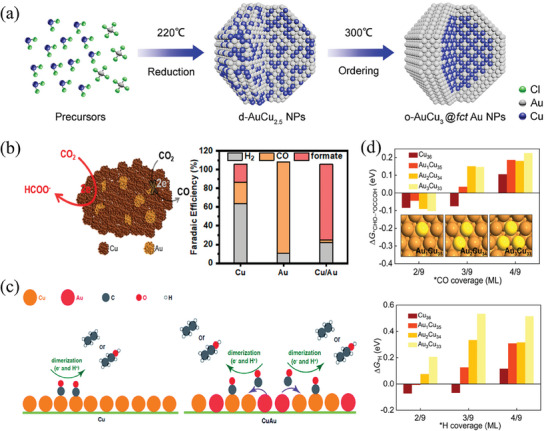
a) Synthesis and structure of o‐AuCu_3_@fct Au NP.^[^ ^167^ ^]^ Copyright 2021, American Chemical Society. b) CO_2_ reduction diagram on Cu/Au catalyst and FE distribution of CO_2_ products on Cu, Au, and Cu/Ag in 0.5 m KHCO_3_ aqueous solution saturated with CO_2_.^[^ ^168^ ^]^ Copyright 2019, American Chemical Society. c) The formation mechanism of C_2_H_4_ and C_2_H_5_OH on Cu and CuAu.^[^ ^171^ ^]^ Copyright 2019, The Royal Society of Chemistry. d) DFT results. The reaction‐free energy difference between the *CO protonation and C‐C coupling steps under different *CO coverages and the reaction‐free energy formed by *H under different *H coverages on the surfaces of Cu_36_, Au_1_Cu_35_, Au_2_Cu_34,_ and Au_3_Cu_33_.^[^ ^173^ ^]^ Copyright 2019, Springer Nature.

During the CO_2_ electrochemical reduction reaction, Cu‐Au alloy foil is effective in significantly increasing CO yield, whereas nanoparticles demonstrate superior performance. The optimization of the size,^[^ ^164^ ^]^ atomic distribution,^[^ ^165^ ^]^ composition, and surface morphology^[^ ^166^ ^]^ of Cu‐Au nanoparticles have significantly enhanced the CO selectivity to 90%. Recently, Yu and colleagues^[^ ^167^ ^]^ successfully applied the concept of interface strain stable metastable phase to produce a metastable face‐centered tetragonal Au‐coated nanoparticle (o‐AuCu_3_@fct Au NP) as presented in **Figure** **11**a. The nanoparticle exhibited an optimal Faraday efficiency of 94.5% for CO at an electrode potential of −0.8 V (vs RHE) and displayed high catalytic stability over a period exceeding 20 h. Furthermore, based on DFT calculations and SR‐FTIR measurements, it has been found that the movement of the d‐band center is more significant in face‐centered tetragonal Au than in face‐centered cubic Au. As per the d‐band theory, this shift causes more anti‐bonding states to rise above the Fermi level and thus facilitate CO production by reducing the reaction energy barrier.

HCOOH was also found to be produced in substantial amounts at low potentials catalyzed by Cu‐Au nanoparticles. The nanoparticle catalysts reported by Tao et al.^[^ ^168^ ^]^ can reach 81% HCOOH Faradaic efficiency (Figure 11b). The activity and selectivity are 15 times and 4 times higher than those of Cu, respectively. The characteristic activity of Au nanoparticles for selective CO_2_ reduction to CO disappears after forming a heterojunction with Cu, and the interaction between Cu and Au stabilizes Cu^1+^ on the surface under reducing potentials. These two factors are closely related to the enhanced selectivity of HCOOH. Another study^[^ ^169^ ^]^ explained the formation mechanism of HCOOH. The formation energies of *COOH and *OOCH on Au (211) are similar, but the unfavorable positive free energy for *H inhibits the formation of the C‐H bond. On the contrary, the Cu (211) surface is favorable for the formation of *OOCH and *H, and this process can occur at low potential. Therefore, Au promotes the formation of *OOCH, and Cu is the active site for the selective production of HCOOH.

Au‐Cu heterostructure catalysts can selectively convert CO_2_ to high‐value chemicals by tandem catalysis.^[^ ^170^ ^]^ The weak adsorption of *CO by Au increases the coverage of *CO on the surface of Cu, which reduces the adsorption sites of *H and inhibits the formation of hydrogen. Gao et al.^[^ ^171^ ^]^ designed an electrode consisting of Au nanoparticle‐coated Cu_2_O nanowires. The Faraday efficiency (≈70%) for CO_2_ reduction to multi‐carbon chemicals was over twice that of Cu, reaching a peak at −1.05 V (vs RHE). In contrast to the characteristics of Cu‐Ag to form alkanes, it is difficult to break the C‐O bond on the Au surface thermodynamically, so Cu‐Au heterojunction catalysts show higher selectivity for oxygen‐containing compounds, such as alcohols (Figure 11c). Previously, Jaramillo et al.^[^ ^172^ ^]^ reported an electrocatalyst consisting of Au nanoparticles deposited on polycrystalline copper foil. It exhibited better performance than AuCu alloy in the production of alcohols, and the selectivity of high‐value multi‐carbon alcohols at a low potential is over 100 times higher than that of CH_4_ and CH_3_OH. The study suggested that the hydrophobic Au on the Cu surface may tend to retain the C‐O bond, thereby forming a *CH_x_OH intermediate, which undergoes further reactions to form a polyol.

It has been reported that Au atoms are beneficial to the protonation of *CO on the Cu surface instead of C‐C coupling at low *CO coverage. This leads to a significant increase in selectivity toward CH_4_. Siton et al.^[^ ^173^ ^]^ constructed Cu‐Au nanofiber electrocatalysts with a surface Au atomic percentage of 7% and observed that the selectivity of the catalyst varied greatly with different current densities. At low current density, the electrochemical reduction reaction primarily generates C_2_H_4_ as the main product. However, the Faraday efficiency of CH_4_ significantly increases at high current density and can reach a maximum value of 56%. DFT calculations revealed that the *CO intermediates on the Au‐Cu surface tend toward hydrogenation reactions with the decrease of *CO concentration (Figure 11d). This phenomenon is more pronounced on the Cu surface at lower *CO concentration. In addition, the formation energy of *H intermediate on the Au‐Cu surface at a high *H concentration is positive, inhibiting the hydrogen evolution reaction.

### 5.3 Cu‐Zn Heterojunction Catalyst

The Zn (101) crystal plane exhibits strong stability and adsorption capacity for COOH, significantly inhibits the hydrogen evolution reaction (HER), and results in higher selectivity for CO.^[^ ^174^ ^]^ Compared with Ag and Cu, the low cost of Zn makes it widely used in preparing catalysts combined with Cu. However, the active chemical properties make it easy for an oxide layer to form on the surface when exposed to air. In addition, the diffusion of the Zn element at the Cu/Zn heterojunction also results in uncertain selectivity for such catalysts.^[^ ^175^, ^176^, ^177^ ^]^

**Figure 12:**
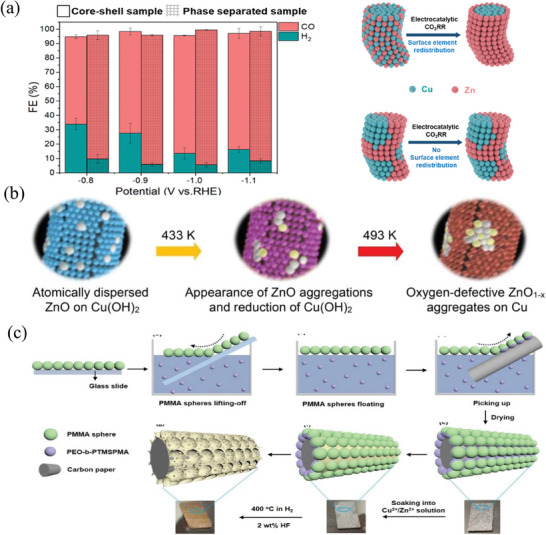
a) The FE distribution of the main products of the core‐shell and phase‐separated samples in CO_2_‐saturated 0.1 m KHCO_3_ aqueous solution.^[^ ^179^ ^]^ Copyright 2022, American Chemical Society. b) A schematic diagram of the structural evolution of the separated ZnO to small aggregates with increasing temperature during the CO_2_ hydrogenation reaction.^[^ ^184^ ^]^ Copyright 2022, Wiley‐VCH. c) Synthesis of HMMP Cu/Zn alloy catalysts on carbon paper by interfacial self‐assembly.^[^ ^186^ ^]^ Copyright 2022, Elsevier.

Cu‐Zn heterojunctions are known to catalyze the reduction of CO_2_ with high selectivity to CO, and dendritic Cu‐Zn catalysts have achieved a Faraday efficiency of 83%.^[^ ^178^ ^]^ In some cases, Cu components with a rich interface structure exhibit higher eCO_2_RR activity, resulting in improved selectivity of the catalysts for CO. Wan et al.^[^ ^179^ ^]^ explored the relationship between the interface structure of the catalyst and the two‐phase interaction of the metal. They prepared CuO_x_/ZnO nanowire catalysts with both phase separation and core‐shell structures (**Figure** **12**a). In contrast, the core‐shell samples displayed poor activity and low FE_CO_, whereas the phase‐separated samples exhibited high selectivity (FE = 94%) for CO. DFT simulation results show that the superiority of the phase separation structure is that it has the lowest energy barrier to form * COOH, which is the rate‐determining step of CO formation. When *COOH was adsorbed on the copper site, the surrounding charge depletion and accumulation regions enhanced the adsorption effect and strengthened the Cu‐C covalent bond. Moreover, the free energy required for the adsorption of hydrogen onto the phase separation structure was relatively high, resulting in lower hydrogen evolution reaction activity.

The selectivity of CH_4_ is significantly decreased due to the segregation of Zn at the Cu‐Zn interface and the CuZn alloy produced in the synthesis process of Cu‐Zn nanoparticles. In a study of Cu_100‐x_Zn_x_ nanoparticles, it was found that the composition of the catalyst and alloy determined the selectivity of the products to a certain extent.^[^ ^180^ ^]^ Through analysis of the XPS energy spectrum and EXAFS data fitting, the initial form of Zn in the obtained sample was found to be ZnO. This ZnO was continuously reduced and alloyed with Cu during the eCO_2_RR process. When the effect of Cu‐Zn is not significant, the Cu/ZnO nanoparticle exhibited high CH_4_ selectivity. When the Zn content was 30%, the highest FE_CH4_ was ≈70%. At this point, the HER reaction inhibition effect was the most obvious, and the *H intermediates were used more for CO_2_ reduction. However, when the Zn content was greater than 70%, the role of the alloy became dominant, and the products were mainly CO and H_2_, and the total Faraday efficiency was ≈90%. Varandili et al.^[^ ^181^ ^]^ found that this phenomenon was related to the composition of the core shell. The effects of ZnO@Cu and Cu@ZnO on the catalytic reduction of CO_2_ were quite different. ZnO@Cu reduced CO_2_ to C_2_H_5_OH, while Cu@ZnO showed the highest Faraday efficiency for CH_4_. The metals on the surface of the two catalysts were also found to be alloyed to a certain extent under eCO_2_RR conditions, and ZnO@Cu with higher Zn content had a higher alloying degree. This mechanism was explained by DFT calculation. The Zn element regulated CH_4_ selectivity by changing the electronic structure of the catalysts. With an increase in Zn content, the d‐band center of the Cu adsorption site gradually moved down, and the formation energy of *COH decreased, reducing the production of CH_4_ on Cu active sites.

Zn has a high oxygen affinity and a strong interaction with the O atom of the CO_2_ molecule, which is conducive to the formation of C‐O.^[^ ^182^ ^]^ However, Zn shows opposite characteristics. Hence, similar to Au, the catalysts formed by ZnO and Cu show high selectivity to alcohols. The inherent characteristics of Zn with a high hydrogen evolution overpotential promote the occurrence of such reactions. While many works have pointed out their excellent performance in CH_3_OH production, the specific role of ZnO in this reaction process remains unclear. Chen et al.^[^ ^183^ ^]^ discovered that the Cu/(Cu + Zn) molar ratio had a significant impact on the formation of oxygen vacancies in the catalysts. The catalytic efficiency of the catalyst reached its peak when the amount of Cu and Zn was equal. At this ratio, the oxygen vacancies in the catalyst played a stabilizing role in the intermediates and facilitated the hydrogenation of *CO to CH_3_OH. The results of Liu et al.^[^ ^184^ ^]^ showed that atomically dispersed ZnO tended to form aggregates rich in oxygen vacancies rather than alloys (Figure 12b). Under the promotion of oxygen vacancies, methanol may be generated through the formic acid pathway (PES) or the water gas shift pathway (RWGS). DFT calculations were used to investigate the four interface structures in the reaction: Cu, CuZn, ZnO/Cu, and ZnO_1‐x_/CuO. The energy barriers for CH_3_OH formation via both pathways at the Cu, CuZn, and ZnO/Cu interfaces were high, whereas the ZnO_1‐x_/CuO interface exhibited high activity for the HCOOH to CH_3_OH pathway. The microkinetic simulation also confirmed that the ZnO_1‐x_/CuO interface was the active site for CH_3_OH production.

Zn can promote the formation of C_2+_ by tandem catalysis in Cu‐Zn heterojunction catalysts, similar to Ag and Au. In a study, Li et al.^[^ ^185^ ^]^ compared the performance of CuO/ZnO/C heterojunction catalyst synthesized by co‐precipitation with ZnO‐CuO/C and CuO‐ZnO/C prepared by sequential precipitation. Both two catalysts showed high CO selectivity at −0.62 V versus RHE, and the amount of CH_4_ and C_2+_ products increased significantly with potential increase. CuO/ZnO/C exhibited the highest selectivity for C_2+_ products, especially C_2_H_4_, with a Faraday efficiency of 71% (−0.74 V vs RHE), while the CH_4_ selectivity was the lowest. This was attributed to geometric effects. The alternating arrangement of CuO and ZnO nanoparticles in the phase‐separated CuO/ZnO/C catalyst can more effectively capture CO on the Cu surface. Conversely, in the other two interconnected nanoparticle matrices, the *CO coverage is insufficient to cause dimerization, instead leading to further hydrogenation to form CH_4_. Later, Su et al.^[^ ^186^ ^]^ demonstrated that hierarchical pores in a Cu_5_Zn_8_ catalyst can increase the concentration of *CO on Cu and prolong its residence time, thereby promoting the production of C_2+_ liquid products at moderate overpotentials (Figure 12c). The Faraday efficiency of the porous Cu_5_Zn_8_ catalyst was 58.3% for C_2_H_5_OH and CH_3_COOH.

The presence of Cu can improve the catalytic performance of Ag, Au, Zn, and other metals with high *COOH adsorption strength for C_1_ products. The electronic effect and microstrain effect at the interface can improve the formation energy barrier of *H and reduce hydrogen evolution by adjusting the electronic structure of the catalysts.^[^ ^187^ ^]^ Nevertheless, the selectivity of the heterostructure catalysts is complicated. Generally, the catalysts display a higher selectivity for CO, while Cu/Ag and Cu/Au nanoparticles can also show higher selectivity for HCOOH at low potentials due to the synergistic effect of surface atoms. ZnO/Cu and Au/Cu catalysts have great advantages in the production of alcohols.^[^ ^188^, ^189^, ^190^ ^]^ The formation of CH_4_ is closely related to the coverage ratio of *CO. The low or high *CO coverage caused by the low content of Ag, Au, and Zn and the high current density is favorable for the formation of CH_4_. When the coverage of *CO reaches a certain range, the heterojunction formed by Cu and another metal can promote C‐C coupling through tandem catalysis. Moreover, the confinement effect and micro‐environmental effect increase the concentration of local *CO, which can better promote the formation of C_2+_ products. At the same time, the inherent characteristics of metals show selective differences in C_2+_ hydrocarbons and C_2+_ alcohols.

## 6 Summary and Outlook

The electrocatalytic reduction of CO_2_ to small molecules is a promising method for converting CO_2_ into useful energy resources. Cu‐based bimetallic heterojunction catalysts, which incorporate the beneficial properties of both Cu and other metals, are capable of efficiently converting CO_2_ into C_1_ products such as CO and CH_4_ as well as high‐value C_2+_ compounds, making them a subject of widespread interest. This review provides a systematic summary of the representative works on bimetallic heterojunction catalysts, which consist of Cu and three other metals, in the eCO_2_RR application. Emphasis is placed on how the interaction between metals and Cu affects the binding strength of intermediates on the catalysts and the selectivity of electrocatalytic reduction products.

**Figure 13:**
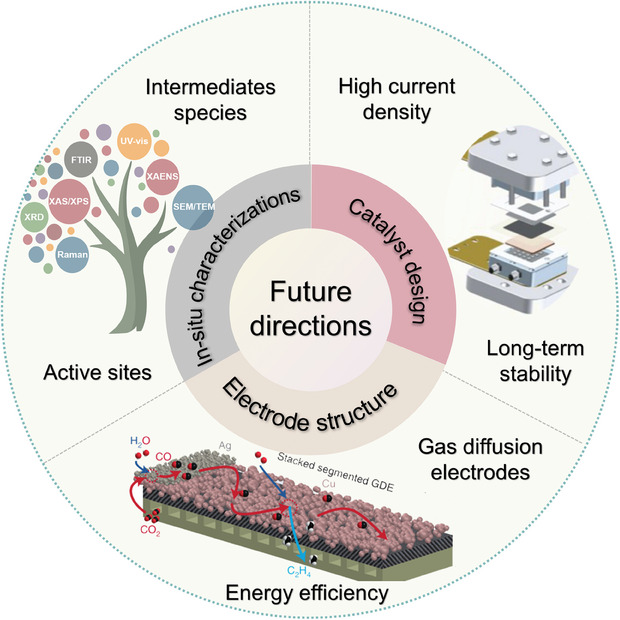
Proposed perspectives and underlying strategies to improve the performance of Cu‐based bimetallic heterojunction catalyst for eCO_2_RR and large‐scale commercialization.

For all three groups of metals, the addition of metallic copper alters the original tendency of these metals to adsorb specific intermediates. Accordingly, these metals improve the poor selectivity of Cu‐based catalysts. When constructing Cu‐based bimetallic heterojunction catalysts, it is crucial to maintain a suitable *CO coverage and balance the adsorption strengths of various reaction intermediates. By adjusting the relative metal content and oxidation state, the geometrical and electronic effects of Cu‐based bimetallic catalysts’ heterojunction can be altered, enabling the modulation of intermediate adsorption. This modulation fully unleashes the catalytic potential of Cu‐based bimetallic heterojunction catalysts for the selective reduction of carbon dioxide. Despite significant progress in the field, there remain significant challenges to the practical application of Cu‐based bimetallic heterojunction catalysts as shown in **Figure** **13**.

1) Enhancing the productivity and durability of electrocatalysts is crucial for their widespread industrial deployment, yet it presents formidable challenges. Conventional copper‐based heterojunction catalysts often exhibit a limited current density of ≈100 mA cm^−2^, a constraint that caps their potential output. Besides, these catalysts struggle to maintain the requisite stability over the 1000‐h threshold essential for industrial use, due in part to surface reactions during electrolysis. To address these impediments, we advocate for an innovative catalytic framework. The incorporation of dopants into the catalyst matrix enhances the interatomic interactions within the catalyst through mechanisms such as charge transfer or orbital overlap, thereby hindering the migration of specific elements. The adjunct of N‐heterocyclic carbene (NHC) ligands offers a means to fortify the metallic surfaces and finely tune the interfacial characteristics. Additionally, encasing the catalyst with a protective layer can prevent the ingress of atoms into the nanoparticle's core, thus preserving the integrity of the heterojunction catalyst under operational stresses. Moreover, stabilization can be achieved through refinements in the electrolysis setup, while judicious manipulation of electrolysis parameters can rejuvenate active sites. These synergistic approaches are designed to ensure the catalyst's sustained performance and efficacy across practical applications.
2) Given the complexity of the catalyst's dynamic evolution during the reaction, the initial structural characterization of CO_2_RR catalysts provided by some non‐in situ techniques may not accurately describe the true active site and its structure‐activity relationship. In‐situ/operando studies have revealed the evolution patterns of catalytic active sites during the catalytic process and delved into the relationship between active sites and the intrinsic activity, selectivity, and stability of CO_2_RR. Operando electrochemical scanning tunneling microscopy (ECSTM) and 4D scanning tunneling microscopy (4D‐STEM), operando atomic force microscopy (AFM), etc., can be used to capture dynamic structures and interface evolutions. In addition, to simultaneously monitor the reconstruction process of copper‐based catalyst surfaces and analyze products, researchers can also use ECSTM‐DEMS (electrochemical mass spectrometry) combined technology, or in situ surface‐enhanced infrared absorption spectroscopy (SEIRAS) and time‐resolved surface‐enhanced Raman spectroscopy (TR‐SERS).

For an in‐depth study of changes in catalyst chemical states and coordination environments, operando high‐energy‐resolution fluorescence detected X‐ray absorption spectroscopy (HERFD‐XAS) is an effective method to provide more detailed data support. In addition, isotopic tracer methods have been used for active site determination and mechanistic characterization. By closely linking structural evolution with changes in product selectivity and combining theoretical calculations, researchers can effectively extract relevant descriptors of the intrinsic structure‐function relationship, thereby gaining a deeper understanding of the catalytic mechanism and optimizing catalyst performance.

3) New electrode structures hold great potential for increasing the current density of electrocatalytic reactions, however, previous studies have mainly focused on catalyst preparation while neglecting electrode design. Gas diffusion electrodes (GDEs) are an example of how electrode design can enhance CO_2_ mass transfer to the active electrocatalyst, resulting in higher energy efficiency and current density. Layered or segmented designs can improve Faraday efficiency and reaction current density for C_2+_ products by regulating CO partial pressure distribution in the thickness and lengthwise directions, guiding for designing other electrodes.

## Conflict of Interest

The authors declare no conflict of interest.

## Data Availability

The data that support the findings of this study are available from the corresponding author upon reasonable request.
